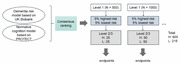# Risk stratification for the enrichment of potential high‐risk groups in a cognitive normal study population

**DOI:** 10.1002/alz70858_102630

**Published:** 2025-12-25

**Authors:** Jonas Botz, Anna‐Katharine Brem, Zunera Khan, Spiros Nikolopoulos, Dag Aarsland, Holger Fröhlich, PREDICTOM Consortium

**Affiliations:** ^1^ Fraunhofer SCAI, Sankt Augustin, NRW, Germany; ^2^ University Hospital of Old Age Psychiatry, University of Bern, Bern, Switzerland; ^3^ King's College London, London, United Kingdom; ^4^ Department of Old Age Psychiatry, King's College London, London, london, United Kingdom; ^5^ Institute of Psychiatry, Psychology and Neuroscience, King's College London, London, United Kingdom; ^6^ Centre for Research & Technology Hellas, Thessaloniki, Greece; ^7^ UK Dementia Research Institute, Institute of Psychiatry, Psychology & Neuroscience, King's College London., London, United Kingdom; ^8^ Centre for Age‐Related Medicine, Stavanger University Hospital, Stavanger, Stavanger, Norway; ^9^ Bonn‐Aachen International Center for IT (b‐it), Bonn, Germany; ^10^ Fraunhofer Institute for Algorithms and Scientific Computing SCAI, Sankt Augustin, Germany

## Abstract

**Background:**

Dementia, particularly Alzheimer's disease, poses a significant health challenge worldwide. The PREDICTOM study aims to address this issue by establishing a comprehensive screening platform for dementia, with a specific focus on Alzheimer's disease. This innovative approach not only seeks to improve early detection but also evaluates novel biomarkers for their predictive value in assessing dementia risk.

**Method:**

The study utilizes a three‐tiered funneling process: Level 1 consists of at‐home screening; Level 2 encompasses a risk assessment that could potentially be carried out by general practitioners; and Level 3 entails a more comprehensive examination, which is usually conducted by a medical specialist. Following the FDA's enrichment trial concept, the screening phase targets the statistical overrepresentation of potential “high‐risk” patients for further assessment in levels 2 and 3. For this purpose, we develop two risk models using retrospective data. The first is a machine learning model based on the UK biobank, estimating individual risk of developing dementia within 3‐5 years post‐baseline, utilizing common risk factors identified by the Lancet commission. The second model, a normative model based on the existing PROTECT study cohort, analyzes age‐dependent cognitive decline (via the FLAME test battery) to assess individual abnormal cognitive function. Both models will be applied to the PREDICTOM participants and subsequently can be used to rank them according to their risk of developing dementia and/or of already showing signs of cognitive decline. To combine the predictions from these models, we will apply statistical consensus ranking using the FAST algorithm.

**Result:**

We will designate the top *n* = 400 patients as the “potential high‐risk” group for Level 2 inclusion, while the bottom *n* = 215 patients will serve as the “potential low‐risk” group. Due to logistical considerations, both groups will undergo Level 2/3 assessments in batches, culminating in Level 3 evaluations for amyloid beta positivity and cognitive impairment (Figure 1).

**Conclusion:**

The risk stratification ensures an enrichment of the potential high‐risk group, allowing for representative analyses of the novel biomarkers and a subsequent multi‐modal analysis accordingly. The results of this study could potentially lead to more effective screening and early intervention strategies for dementia.

**Acknowledgment:**

This Project Is Supported By The Innovative Health Initiative Joint Undertaking (IHIJU) Under Grant Agreement No101132356. The JU Receives Support From The European Union's Horizon Europe Research And Innovation Programme. This Work Was Funded By UK Research And Innovation (UKRI) Under The UK Government's Horizon Europe Funding Guarantee[UKRI Reference Number:10083181]. In Switzerland The University Of Geneva Is Funded For PREDICTOM By The Swiss State Secretariat For Education Research And Innovation (SERI‐ Ref‐1131 52304).